# ‘Them’ without ‘us’: negative identities and affective polarization in Brazil

**DOI:** 10.1080/2474736X.2022.2117635

**Published:** 2022-09-05

**Authors:** João Areal

**Affiliations:** Mannheim Centre for European Social Research (MZES), University of Mannheim, Mannheim, Germany

**Keywords:** affective polarization, negative partisanship, political identities, brazilian politics

## Abstract

High levels of hostility between those on opposing sides of politics have led to a burgeoning literature on the concept of affective polarization. Though a globally widespread phenomenon, extant literature has generated theoretical expectations and empirical findings mostly inspired by the United States and Western Europe. By studying the case of Brazil, I argue and show that traditional explanations do not provide satisfactory accounts of affective polarization in contexts where politics is only weakly structured by ideology or partisan attachments. I argue and show that in such contexts the concept of negative political identities can provide a much better explanation for why politics is so divisive. Using both the 2014 and 2018 waves of the Brazilian Electoral Studies (BES) and independently collected survey data (*N* = 1732), I provide robust empirical findings supporting the primacy of negative political identities over traditional explanations. Negative identification with the out-party/leader has a strong effect on dislike towards out-voters even when controlling for instrumental evaluations of political elites. This paper contributes to the comparative research agenda on affective polarization outside Western contexts, as well as to the study of negative political identities.

## Introduction

After years of mass protests, economic crises and corruption scandals, scholars and journalists often remark that Brazil is engulfed in deep political polarization (e.g. Borges 2021; Mignozetti and Spektor 2019). The 2018 electoral success of the far-right president Jair Bolsonaro, amidst a campaign filled with rash rhetoric and hostility between political opponents, has epitomized the divisive nature of Brazilian politics. Politics has come to divide friends and families like perhaps never before in Brazil, and polarization shows no signs of abating: Brazilians of different sides of the political spectrum dislike each other to increasing and alarming levels. Understanding *why Brazilians dislike their fellow citizens on the opposite side of the political divide* is the principal goal of this paper.

This animosity between political opponents has been termed *affective polarization*, understood here as inter-citizen political hostility. This hostility between individuals from different sides of politics has been shown to have pernicious effect for social integration and democracy at large (Carothers and O'Donohue 2019), with a vast body of work dedicated to understanding the factors that turn politics into a nasty game between enemies. The bulk of extant literature on affective polarization, however, is concentrated either on the specific cases of the United States (Iyengar et al. 2019) or Western Europe (e.g. Wagner 2021), which present particular features. Such contexts are characterized by highly institutionalized party systems (Dalton and Weldon 2007), programmatic patterns of political competition (Kitschelt 2000), strong linkages between social groups and political parties (Lipset and Rokkan 1967), and entrenched and stable partisan identities (Huddy, Mason, and Aarøe 2015).

Like most of Latin American (Kitschelt et al. 2010) and Eastern European (Rose and Mishler 1998) countries, Brazil displays none of the above features. Brazilian politics is only weakly structured by ideology (Oliveira and Turgeon 2015), stable partisan identities have largely failed to take root (Samuels and Zucco 2018), and parties lack clear linkages with distinct social groups (Zucco and Power 2020). These considerations present a significant challenge to traditional mechanisms, which posit that people dislike each other either because they hold vastly contrasting views, or because they are deeply attached to their respective ‘side’ of politics. By systematically operationalizing and testing these classic explanations for the first time in Brazil through analyses of the 2014 and 2018 waves of the Brazilian Electoral Study (BES), I show that these lines of explanation perform particularly poorly in the Brazilian context. Studying the case of Brazil, therefore, is of significant importance to the broader field of affective polarization not simply because it is yet another country suffering from acrimonious political divides. Rather, understanding how affective polarization can take place through non-traditional ‘sources’ can significantly advance our understanding of this phenomenon.

If not through ideology or strong partisan identities, how can politics be so divisive? I build on the concepts of negative partisanship (Medeiros and Noël 2014; Samuels and Zucco 2018) and negational identities (Zhong et al. 2008), and propose a theoretical framework underpinning the notion of *negative political identities* in order to explain affective polarization in Brazil. Negative political identities emerge independently out of a strong rejection of the opposing side's party or political figurehead, and can exert strong independent effects on political behaviour. By leveraging independently collected survey data (*N* = 1732) amongst Brazilian respondents, I put forward an innovative empirical measure of negative political identities. I show that a deep rejection of the the opposing side's party/leader constitutes a prevalent form of social identity (e.g. *antipetista* and *antibolsonarista*), and that these negative identities are the primary mechanism through which Brazilians dislike their fellow citizens. These findings are robust to models that control for negative instrumental evaluations of the out-party/leader, isolating the independent effects of negative political identities in generating out-voter dislike. Further, the effects of negative identities far surpass those of ideological disagreements and in-group attachments, indicating that Brazilians dislike ‘them’ even without a clearly defined sense of ‘us’.

The strength of negative political identities amongst the Brazilian electorate bespeaks an increasingly hostile and uncompromising polity, which should worry political practitioners and scientists. As citizens turn away from parties (Dalton and Wattenberg 2002), negative political identities are an increasingly important factor structuring political competition both in new (Haime and Cantú 2022) and advanced democracies alike (Bankert 2021). The theoretical and empirical contributions on the concept presented by this paper should thus be of significant relevance to the study of comparative political behaviour.

## Theoretical framework

### Affective polarization: when adversaries become enemies

Political conflicts have always been at the heart of liberal democracies, and competing political agendas are thought to be a hallmark of a healthy competitive polity (Mainwaring 2018). Over the past few years, however, US scholars have noted that Americans from different sides of politics do not simply disagree — they increasingly fear, distrust, and even hate each other (e.g. Iyengar, Sood, and Lelkes 2012; Mason and Wronski 2018; Webster and Abramowitz 2017). These developments have led to a burgeoning literature on the concept of *affective polarization*, describing the levels of hostility individuals feel towards the those from the ‘other side’ of politics. Though initially restricted to the United States, more recent works by comparative scholars have translated the concept of affective polarization to multi-party systems in Europe, highlighting that the US is not alone in its levels of political divisions (Harteveld 2021a; Hobolt, Leeper, and Tilley 2020; Wagner 2021).

I formally define this dislike towards the political ‘other’ as *hostility towards political out-groups*. This definition captures animus between partisan (Webster and Abramowitz 2017), ideological (Mason 2018a), social (Harteveld 2021b) or even issue-based (Hobolt, Leeper, and Tilley 2020) groups.^1^ A second necessary clarification is that affective polarization is understood here as an *inter-citizen phenomenon*. Past research has shown that individuals' feelings towards political parties are significantly different to feelings towards their fellow citizens (Druckman and Levendusky 2019; Kingzette 2021), and might be capturing general ‘anti-politics’ feelings amongst politically disillusioned voters (Klar, Krupnikov, and Ryan 2018). Since Brazilian parties lack clear social bases of support and tend to be highly distrusted by the electorate (Bargsted, Somma, and Castillo 2017), it is all the more likely that Brazilians' attitudes towards parties or political elites might not capture inter-citizen hostility.

### Affective polarization in Brazil

While, to the best of my knowledge, there are no published works that attempt to study these divides through the lenses of affective polarization at the time of writing, it is widely acknowledged that Brazil is engulfed in polarization since at least 2014 (Mignozetti and Spektor 2019). A combination of corruption scandals and a deep economic recession led to growing dissatisfaction with the incumbent left-wing Workers' Party (henceforth PT). A bitterly divisive impeachment process ensued, with the 2016 removal of then president Dilma Rousseff from office decried as a *coup d'état* by the PT and its supporters. With much of the political class discredited by their association with corruption scandals, far-right populist Jair Bolsonaro capitalized on a strong rejection of the PT and emerged as a credible alternative to mainstream parties at the 2018 presidential contest. Though ultimately victorious, Bolsonaro hostile rhetoric and extreme policies antagonized many Brazilians, and both and the PT's presidential candidate had high levels of rejection amongst opposing groups of voters (BBC 2018). In the years since the acrimonious contest of 2018, protests both against and in support of Bolsonaro's government have become commonplace (Folha 2020) and political disagreements have entered people's personal networks (Machado et al. 2019).

### Traditional explanations for affective polarization

In reviewing the extant literature on the individual-level mechanisms behind affective polarization, I start by deriving testable hypotheses that have been employed by scholars in the United States and Western Europe. These mechanisms are broadly based on ideological disagreements and conflicts arising out of individual's attachment to socio-political groups. After presenting each hypothesis I reflect on their applicability to Brazil, arguing that, owing to the weak ideological structuration of Brazilian politics and low levels of partisan identification amongst the electorate, mainstream explanations are unlikely to provide a satisfactory account of affective polarization in Brazil. I thus propose an alternative hypothesis couched on the idea of *negative political identities*. I hypothesize that Brazilians dislike ‘the other side’ not through ideological disagreements or identification with their ‘own side’, but rather because the opposing party/leader provides them with a *negative identity*. These negative identities, I shall argue, are the primary driver of out-group hostility in Brazil.

#### Ideological roots of affective polarization

Though ideological and affective polarization are theoretically distinct concepts, it is expected that a certain level of hostility should be present if political groups disagree on salient issues (Webster and Abramowitz 2017). American scholars (Lelkes 2021; Orr and Huber 2020) have found that individuals tend to dislike those who they perceive as being too ideologically distant from themselves, though comparative evidence finds only only weak associations between ideological and affective polarization (Gidron, Adams, and Horne forthcoming). Alternatively, Bougher (2017) argues that individuals can dislike ‘the other side’ through the notion of *party-issue constraint* (see Converse 2006). Party-issue constraint captures the extent to which division over issues and policy are structured along partisan lines (Baldassarri and Gelman 2008). Crucially, this party-issue alignment is independent of the overall relative ideological distance between voters: partisan camps might not be so ideologically distant from each other, but, due to strong party-issue alignment, they might disagree on nearly everything. Ideological disagreements can thus fuel affective polarization in at least two ways: *ideological distance* and *party-issue constraint*.

Hypothesis 1a: Higher levels of ideological distance from the opposing side are associated with higher levels of affective polarization.

Hypothesis 1b: Higher levels of party-issue constraint are associated with higher levels of affective polarization.

#### The minor role of ideology in Brazilian politics

Political competition in Brazil, however, is only weakly structured by ideological considerations, standing in sharp contrast to the United States (Baldassarri and Gelman 2008) and Western Europe (Otjes and Rekker 2021). The Brazilian party system is weakly institutionalized (Mainwaring et al. 2018), with parties deriving their support from pork-barrel politics (Ibid.), leader's evaluations (Baker et al. 2016), clientelism (Mainwaring 1999), or the state of the economy (see Carreirão and Renno 2018 for a review). Given the role of political elites in both structuring (Levendusky 2010) and polarizing (Zechmeister 2015) the electorate, it is unlikely that ideology should meaningfully divide voters. Indeed, several past studies (e.g. Borges and Vidigal 2018; Oliveira and Turgeon 2015) have found little ideological differentiation between partisan camps, with issue positions being only weakly (if at all) related to vote choice or party affect. Similarly, Brazilians are said to lack a basic understanding of left and right and to show low levels of issue constraint (Ames and Smith 2010; Kearney and Machado 2018). Both these considerations directly weaken Hypotheses 1a and 1b: if politics is not fought along ideological lines, ideology should hold little explanatory power for affective polarization in Brazil. Indeed, this speaks to comparative evidence by Reiljan (2020), who finds a weak association between ideological and affective polarization in the weakly institutionalized party systems of Eastern Europe.

A valid counter-argument here is that elite-level polarization has been on the rise (Zucco and Power 2020), further compounded by the emergence of far-right president Jair Bolsonaro, who may have ideologically polarized the electorate (Zechmeister 2015). Indeed, Amaral (2020) reports a growing number of Brazilians placing themselves at the far-right end of the ideological spectrum. However, past research has consistently found ideological self-placement to be highly endogenous to presidential candidate affect (e.g. Baker et al. 2016; Borges and Vidigal 2018), such that the rise in the number of ‘far-right’ Brazilians might be an artefact of a growing identification with Jair Bolsonaro rather than a meaningful change in ideological beliefs.

#### The interplay of social identities

While acknowledging the effects of ideological disagreements, the bulk of the affective polarization literature has emphasized the role played by social identities (Iyengar et al. 2019). This approach is couched in the Social Identity Theory (SIT) (Tajfel et al. 1979), which posits that individuals derive a sense of identification from social groups to which they belong. This sense of identification fosters a categorization of the world into ‘us’ (in-group) versus ‘them’ (out-group), even if group differences are minimal or trivial. Building on the SIT framework, political scientists have put forward a conceptualization of partisanship as a social identity itself (Greene 2002). Much like individuals may define themselves based on race, religion or class, belonging to a particular political camp might also inform one's sense of self.

Social identities provide a powerful framework for explaining affective polarization. Firstly, identifying with an in-group may foster out-group hostility as individuals seek to maintain their own group's positive image and higher status (Abrams and Hogg 1988). This is particularly the case for strong identifiers, to whom gains and losses are an emotional, personal matter (Huddy, Mason, and Aarøe 2015). Indeed, a large body of literature has found strong evidence for the effects of in-group attachments on affective polarization, both comparatively (Wagner 2021) and in single-country studies (Dias and Lelkes 2021; Hobolt, Leeper, and Tilley 2020; Mason 2016).

Secondly, out-group hostility is also likely to arise when several group identities — such as race, class and partisanship — overlap (Roccas and Brewer 2002), a phenomenon called *social sorting* (Mason 2016). For example, if the majority of white and Protestant voters tend to identify with the Republican party, a white, Protestant Republican partisan will have their political, social and religious identities ‘aligned’ on the same side of the political divide. Socially sorted individuals are less able to cope with threat than those with cross-cutting identities (Ahler and Sood 2018; Mason and Wronski 2018), with evidence from the US (Mason 2018a) and cross-country comparisons (Harteveld 2021b) providing evidence for the link between social sorting and affective polarization.

We should thus expect high levels of party identification and social sorting to be significant and substantial predictors of affective polarization:

Hypothesis 2a: Stronger partisan identification is associated with higher levels of affective polarization.

Hypothesis 2b: Higher levels of social sorting are associated with higher levels of affective polarization.

### Social identities in Brazil: rare and non-politicized

If the interplay of social identities is the main driver of affective polarization, however, it follows that strong levels of attachments to political in-groups must exist amongst a significant part of the electorate, playing an important role in structuring how citizens engage with politics. This is not the case in Brazil, however, where partisan attachments are not widespread nor linked to politicized social identities.

Firstly, the number of partisans in Brazil has been unstable and comparatively low, reaching 50% of the electorate in its peak, but generally hovering at around 30% (Samuels and Zucco 2018). Partisan attachments are primarily connected to leaders' personal appeals and party performance rather than a strong sense of identification (Baker et al. 2016), and thus fit poorly within an identity-based framework. In other words, Brazilian partisans are few in quantity and low in terms of social identification, making partisanship an unlikely explanation for out-group hostility in the country.

Secondly, Brazil differs significantly from the United States (Mason 2018b) and Western Europe (Helbling and Jungkunz 2020) in the extent to which social cleavages structure partisan competition. Though racial, income and regional inequalities run deep in Brazilian society, these cleavages have not been strongly articulated into politics, neither have they been properly mobilized by political actors (Samuels and Zucco 2014a). Political parties have seldom tried to convey group-based appeals and have been largely unsuccessful when they did try, resulting in rather heterogeneous social bases of support without clear linkages with specific social groups (Mainwaring 1999). However, relevant exceptions could be made with regards to the PT's association with the Northeast (Hunter and Power 2007), the recent alignment of conservative Evangelicals with the right-wing (Quadros and Madeira 2018; Smith 2019), and the effect of gender and race on presidential vote in the 2018 election (Layton et al. 2021). In spite of these examples, the fact that social identities are generally ‘only weakly politicised at best’ (Zucco and Power 2020, 2) means that, even if some social groups are aligned with a particular partisan camp, the social sorting mechanism is unlikely to be a substantive driver of out-group hostility in Brazil.

In short, mainstream explanations, based on ideology and in-group attachment, are unlikely to produce a satisfactory account of the roots of affective polarization in Brazil.^2^ How, then, can politics be so divisive? I attempt to solve this puzzle through the notion of *negative political identities*. I posit that a strong rejection of the ‘other side's’ party or figurehead constitutes a meaningful form of social identity, and acts as a significant driver of out-group hostility. In the following section, I first outline the mechanisms underlying negative political identities and then situate it in the Brazilian context, where negative identification with the PT (*antipetismo*) or Jair Bolsonaro (*antibolsonarismo*) should provide a much stronger explanation for affective polarization than ideology and positive social identities.

### ‘Them’ without ‘us’: how negative identities fuel affective polarization

The concept of social identities has long been couched in the idea of an individual's belonging or attachment to a particular social group (Tajfel et al. 1979). From this perspective, in-group identification is psychologically prior to the definition — and derogation — of out-groups. Negative feelings and biases towards ‘the other’ must then be fundamentally couched in a prior sense of identification to an in-group. Zhong et al. (2008), however, argue that individuals can also define themselves *negationally*, in terms of who they are *not*, even if no prior in-group identification is present. In the same vein, Leonardelli and Toh (2015) argue for the existence of an ‘out-group-only’ categorization, where an individual perceives a cohesive group as ‘the other’ to which she does not belong, without a corresponding in-group. In theorizing about the psychological roots of negative identities, Zhong et al. (2008) draw on Optimal Distinctivness Theory (Brewer 1991), which posits that social identities satisfy two basic human needs: a need for belonging (to an in-group) and a need for distinctiveness (i.e. differentiating oneself from out-groups). Positive identities can satisfy both needs, and should thus be preferred by individuals. In the absence of in-group belonging, however, negative identities can emerge out of an independent and primary sense of *differentiation from an out-group*. This means that, for instance, individuals might define themselves as ‘not-Republican’ without having any attachment or identification with the Democrats.

Since negative identities are primarily informed by one's need to differentiate oneself from a particular group, they are likely to have significant effects on out-group hostility (Leonardelli and Toh 2015). In fact, Zhong et al. (2008) find that negative identities have a higher effect than positive ones in fostering negative feelings towards individuals associated with the out-group label. Further, a vast literature on the concept of negativity bias (e.g. Soroka 2014) suggests negative information may exert greater effect on attitudes than positive information. I thus derive the central hypothesis in this paper:

Hypothesis 3: Negative identities have a stronger effect on affective polarization than positive identities.

Two crucial points need to be made for a proper test of Hypothesis 3. Firstly, this hostility should stem primarily from a true sense of negative identification with the out-party. People might simply ‘dislike’ a political party for instrumental reasons connected to policies or performance (Huddy, Mason, and Aarøe 2015), without negatively identifying with said party (Bankert 2021). The mechanism behind Hypothesis 3, however, requires that we conceptualize (and measure) negative partisanship as a type of social identity ^3^ couched in the underlying logic that identities and evaluations (or attitudes) are conceptually distinct (Hallajow 2018). A test of Hypothesis 3 should therefore separate out the effects of instrumental evaluations from those that arise out of a sense of negative identification.

Secondly, the out-group being evaluated must be comprised of individuals associated with the party (e.g. voters) rather than the party itself, lest we risk making the merely tautological point that a negative party identity leads to negative evaluations of the out-party. In short, Hypothesis 3 posits that a negative party identity leads to more negative evaluations towards out-party voters when compared with positive identities, net of a mere ‘dislike’ of the out-party.

Why might negative identities emerge in the first place, and, more importantly, why would they apply so well in Brazil? Whilst there is a growing literature on the concept of negative partisanship, the focus is mostly on its behavioural consequences rather than potential causes (e.g. Mayer 2017; Ridge 2020).^4^ Further, with the notable exception of Bankert (2021), extant literature does not embed the concept of negative partisanship within a framework of social identities. In the following sub-section, I attempt to translate the psychological mechanism proposed by Zhong et al. (2008) to the realm of politics, drawing on the case of Brazil for further theoretical and empirical leverage.

#### Towards a theory of negative political identities: antipetismo and antibolsonarismo in Brazil

In the low-partisan context of Brazilian politics, scholars have long emphasized the relevance of *antipetismo*, a strong rejection of the centre-left PT, in structuring voters' behaviour.^5^ As Samuels and Zucco (2018) argue, both positive and negative party attachments in Brazil have long revolved around the PT, as the party's electoral success and deliberate efforts to cultivate a clear brand made it a clear and salient party to either like or dislike. In contrast, the largely non-programmatic alternatives to the PT did not particularly attempt to develop a widespread partisan base (Samuels and Zucco 2014a). This meant that non-supporters of the PT lacked any clear party to belong to and thus could not satisfy their need for in-group belonging. The high salience of their out-party, in turn, fostered a *need for distinctiveness*, the bases of negative identity formation. Indeed, *antipetismo* is not simply a rational negative evaluation of the PT: it has been shown to act as a source for perceptual bias and motivated reasoning (Samuels and Zucco 2018), and, though the PT is the standard-bearer of the Brazilian left, not to be primarily grounded in ideological beliefs (Ribeiro, Carreirão, and Borba 2016; Samuels and Zucco 2018).^6^

If *antipetismo* is a prevalent negative identity, and if negative identities lead to affective polarization, this hints at why Hypothesis 3 might apply so well in Brazil. However, this does not explain why PT sympathizers (or, more broadly, left-leaning voters) might dislike ‘the other side’. I argue that this was due to a lack of a clear out-group for PT sympathizers or left-leaning Brazilians, since no other mainstream party cultivated a partisan base to stand in contrast to that of the PT. The notion of a clear and recent *bolsonarista* identity, however, is far more concrete (Rennó 2020), laying the grounds for a negative *antibolsonarista* social identity. Following Samuels and Zucco (2018)'s argument, positive and negative identities may emerge ‘symbiotically’, with the prevalence of the former informing the latter.

An anti-Bolsonaro identity, however, is not dependent on prior attachment to the PT. Bolsonaro's extreme policies and rash populist rhetoric can foster a need for distinctiveness even amongst voters who did not particularly like the PT or the political left (and thus lack any clear in-group). Bischof and Wagner (2019), for instance, argue that far-right parties can motivate individuals to signal their disapproval for their extreme policies and discourse, fostering a need for differentiation. Similarly, Gidron et al. (forthcoming) show that radical-right parties in Western Europe tend to be more disliked than their ideological extremism would predict. We can couple this policy extremism with Bolsonaro's blatantly hostile and populist rhetoric, which is also theorized to evoke strong negative feelings amongst voters (Meléndez and Kaltwasser 2021). Indeed, Meléndez and Kaltwasser (2021) have recently shown that the Populist Radical-Right party family has the highest number of negative identifiers in Western Europe.

The theoretical underpinnings of Hypothesis 3 should be much clearer now. Contextual political factors — such as lack of efforts on the part of elites to develop partisan identification, non-programmatic political competition, extreme policies and divisive rhetoric — can make in-group belonging less likely for a significant portion of voters, whilst at the same time fostering a need for these individuals to distance themselves from relevant political actors. Through a need for distinctiveness, Brazilians ‘negatively identify’ with the party/leader that symbolizes the ‘other side’ of politics, which is theorized to have strong effects on out-group hostility.

This line of explanation should go well beyond Brazil. Negative partisanship is particularly important for understanding political attitudes in Eastern Europe (Rose and Mishler 1998) and several Latin American countries (Haime and Cantú 2022), contexts in which positive identification with parties is low and political competition is often dichotomized. Further, the rise of negative partisanship in Western Europe (Meléndez and Kaltwasser 2021) and the United States (Abramowitz and Webster 2016) suggests negative partisanship can exert independent effects on political behaviour even in more established, high-partisan polities. Therefore, rather than acting as a unique example, Brazil is one of many potential cases where affective polarization may be better understood through the lenses of negative partisanship rather than strong in-group attachments or ideological disagreements.

## Analyses

Testing the mechanism that links negative political identities and affective polarization presents a significant empirical challenge. Firstly, we need a direct measure of negative identities, which goes beyond affective ratings of parties or politicians that might simply capture instrumental evaluations. Bankert (2021)'s study aside, there is no such measure in any existing dataset, and works on negative partisanship have either used like/dislike scales or question-items indicating which party a respondent would never vote for. Since it would be pure tautology to show that ‘never voting for a party’ is associated with negative feelings towards that party, we also need to operationalize affective polarization as hostility towards other citizens. Affective ratings of voters or other political groups, however, are hard to come by, and literature on affective polarization has typically relied on party affective ratings. A proper test of Hypothesis 3, thus, requires a more fine-grained operationalization of both negative identities and out-group hostility than the ones that are commonly found in existing datasets.

To remedy these issues, I make use of two data sources: the Brazilian Electoral Study (BES) and survey data I collected through an online survey in April and May 2020. This analytic strategy can leverage the high quality of the BES samples and the fine-grained measures provided by my survey data. By ‘triangulating’ the empirical results, findings can be supported by both external and internal validity.

### Data

#### Brazilian Electoral Study (BES)

The Brazilian Electoral Study (BES) is part of the Comparative Study of Electoral Systems (CSES) database and collects measures of political and social attitudes amongst a nationally representative sample of voting-age Brazilians in the weeks following national elections. Though these data have been collected since 2002, the lack of comparability on certain question-items means that model results are restricted to the last two BES waves, encompassing the 2014 and the 2018 elections. The final analytic sample consisted of 2870 respondents.

#### Online survey

The online questionnaire was circulated throughout April and May 2020, reaching a total of 1728 Brazilians aged 18 or above. Participants were asked for their informed consent before the beginning of the questionnaire.^7^ To encourage participation, respondents were given the opportunity to take part in a raffle awarding two gift-cards worth 50 EUR each.^8^ Respondents came from two main sources: personal networks and the online forum *reddit*. My primary mode of distribution through personal networks was by sharing the survey in *WhatsApp* groups, specifically targeting groups that are not related to politics and encompass a diverse demographic profile. *WhatsApp* groups are considerably popular in Brazil, especially amongst lower socio-economic classes (Spyer 2017), and are characterized by a culture of open political discussion amongst participants (Machado et al. 2019). This facilitated the sharing of the online questionnaire on this platform, and I estimate that around 375 respondents were reached via *WhatsApp*. Engagement with the questionnaire was also significant on *reddit*, with around 1353 Brazilian *redditors* taking part in the survey over the course of a week.

Table A7 in the Appendix shows how my sample compares to the BES 2018 sample on key demographics. In short, the online survey respondents were more male, white, of higher income and educational levels, and younger. The biggest source of bias is, by far, education — while only 28% of BES 2018 respondents had some level of tertiary education, this was true for 90% of survey respondents. The sample is also skewed in terms of 2018 vote choice, with only 23% (*n* = 289) of reported valid votes going towards Jair Bolsonaro (in contrast to the actual 55% vote share at the election).

#### Addressing sample bias

To account for some sources of unrepresentativeness, I apply weights based on gender, race, education, income, region of residence, and self-reported political interest. I use the BES 2018 rather than national census data as a proxy for the true population values in order to weigh the sample by political interest, which may act as a proxy for political sophistication, a variable found to moderate the effects of political attitudes in Brazil (Nicolau 2014).^9^ The high skewness of certain weighting variables (in particular, education), however, means that respondents from underrepresented categories might receive extremely high weighting scores, exerting undue influence on empirical results. To remedy this issue, I apply lower and upper constraints on the survey weights, such that no respondent has a weight lower than 0.3 or higher than 3.^10^

This does mean that the weighting procedure cannot satisfactorily account for sample bias introduced by the high baseline levels of education in my sample. I address this issue by interacting education with all independent variables inserted into the following BES model (i.e. ideological distance, issue constraint, social sorting and partisanship strength), and find no statistically significant effects. This indicates that, at least when other variables and demographics are controlled for, highly educated individuals do not behave any differently when it comes to the hypothesized relationships. A second consideration on sample bias is that the survey respondents do show significant variation in their self-reported levels of political interest. Political interest should capture much of the biases introduced by high levels of education, and the variation in responses allows weights to reasonable account for over-representation of highly educated respondents. In sum, though unrepresentative of the Brazilian population, the survey data should allow for relatively robust tests of the mechanisms under analysis.

### Operationalisation

#### Dependent variable

For the BES data I operationalize affective polarization as the reverse-coded affective rating (ranging from 0 to 10) respondents attributed to the party they did not vote for in the second round of presidential elections.^11^ Second-round voting captures the most relevant lines of political competition in Brazil, pitting the centre-left PT against right-wing and centre-right competitors since 1989 (Mello and Spektor 2018).

As previously alluded to, feelings towards parties may capture dislike towards party elites rather than voters, a pertinent issue in Brazil where parties are generally distrusted by the electorate. Through the online survey I captured affective ratings (using the same 0–10 scale) towards parties, partisans and voters. This allows both a proper test of Hypothesis 3 (which links negative out-party identification with out-voter dislike), as well as a more fine-grained measure of affective polarization. In the following analyses I use out-voter dislike as the dependent variable so as to separate feelings towards elites from those directed at citizens, which can alleviate endogeneity problems in the proposed relationship (i.e. negative identification and out-group dislike).^12^

#### Independent variables

##### Measures of positive and negative identities

In the BES data I measure *partisanship* (positive political identity) through a 3-item question ranging from ‘low’ to ‘strong’. This results in a categorical variable, with nonpartisans serving as the baseline category. No measures of negative identification were present, and thus Hypothesis 3 cannot be tested with BES data.

In the online survey I use a more fine-grained measure of partisan identities by employing the Party Identity Scale (PID) developed by Greene (2002) and used by and Bankert, Huddy, and Rosema (2017). This scale captures a sense of belonging to an in-group, the salience of such identification, and the effects of lowered group status. Respondents were asked about their levels of positive identification with the party/candidate they voted for in the second round of the 2018 presidential election.

To measure *negative identification* (NID) I reverse the PID scale items in order to capture a sense of differentiation from the relevant out-group, and whether this informs a respondent's self-concept. This ‘reversal’ from the PID scale serves two other purposes. Firstly, it makes the measures directly comparable and thus allows for a robust test of their independent effects (see Hypothesis 3). Secondly, this direct comparability means that the tried-and-tested PID scale can serve as an ‘anchor’: if NID can also be reliably measured amongst the same respondents, this can add robustness to its measurement properties and alleviate sample bias concerns. Accordingly, NID items were asked about the party/candidate the respondent did not vote for.

Table 1 presents the exact wording of the items used to measure both positive and negative identities, with all items ranging from 1 (strongly disagree) to 5 (strongly agree). In the bottom row of the table we can find the Cronbach's alpha, which tests whether these items can be combined into a reliable scale. Both the positive and negative identity scales returned scalability scores above conventional 0.6 cut-off points, with NID even presenting a slightly higher coefficient than the tried-and-tested PID. This suggests that negative political identities can indeed be reliably measured through survey items, much like their positive counterpart. However, scalability analyses do not tell us whether negative and political identities form independent constructs. Critics could argue that simply measuring a level of positive identification would, to a large extent, also capture the strength of one's negative identity.

**Table 1.** Items measuring positive and negative political identities.

Positive identity scale (PID)	Negative identity scale (NID)
1. When people criticize the PT (Bolsonaro), it feels like a personal insult	1. I feel good when people criticize Bolsonaro (PT)
2. If I know someone voted for the PT (Bolsonaro), I sympathize more with this person	2. If I know someone dislikes Bolsonaro (PT), I sympathize more with this person
3. I have a lot in common with people who voted for the PT (Bolsonaro)	3. People who voted for Bolsonaro (PT) are very different from me
4. I would describe myself as petista (bolsonarista)	4. I would describe myself as anti-Bolsonaro (-PT)
Cronbach's α=0.76	Cronbach's α=0.78

To address these concerns and to establish the empirical independence of negative identities, I proceeded with an exploratory factor analysis of all identity question-items (i.e. the four PID & NID items). In the first step, the *eigen* decomposition procedure suggested the existence of 2 separate factors that account for the variation in the eight items. All NID items loaded strongly on the same dimension and very weakly in the second factor (See Tables A8 and A9 in Appendix), further suggesting that the negative identity scale is internally cohesive and captures an independent latent trait.

Conversely, the second and third PID items in Table 1 had slightly stronger factor loadings on the ‘negative’ dimension and had generally weaker loadings altogether, suggesting a poor fit with the proposed factors. This eight-item, two-dimensional model overperforms a one-dimensional model through a confirmatory factor analysis (see Table 2) but does not meet conventional measures of model fit. Removing items 2 and 3 from the PID scale, however, returns a two-factor model with both RMSEA and RMSR below 0.08, and Tucker Lewis and Comparative Fit Indices above 0.95 (see Table A10).

**Table 2.** Confirmatory factor analysis.

	$\chi^{2}$	df	CFI	RMSEA	TLI
One-Factor (8 items)	902.63	20.00	0.72	0.19	0.61
Two-Factor (8 items)	437.18	19.00	0.87	0.14	0.81

For theoretical reasons I employ the full PID and NID scales in the main models. However, inconsistencies in the PID items may be adding random noise to the scale, potentially masking the impact of positive identities on affective polarization. As a robustness check, I run alternative models where only the fourth item in both scales is used (i.e. ‘I would describe myself as (anti)…’). I return to these results when discussing the model findings.

##### Separating negative evaluations from negative identities

In order to isolate the independent effect of negative identification from instrumental evaluations in the online survey models, I control for *out-party affective ratings*. In short, it means that, even when accounting for how much a respondent simply ‘dislikes’ their out-party or candidate, negatively identifying with it should still return a strong effect on out-voter dislike. Further, this effect should be stronger in size than the effects of positive identification. This strategy places a particularly ‘hard’ control on the negative identity scale, which should provide for more robust results in testing Hypothesis 3.

##### Other independent variables

Hypotheses 1a and 1b require a measure of respondents' ideological beliefs. The traditional left-right scale, however, is ‘notoriously noisy’ in Brazil's non-programmatic context (Samuels and Zucco 2018, p. 40), and has been shown to be highly endogenous to (especially presidential) affect. I thus make use of four question-items in both the BES and online survey data that capture respondents' stances on cultural and economic liberalism axes of political competiton, which have also been broadly reflected in the agendas associated with the PT and its right-wing competitors (Ribeiro, Carreirão, and Borba 2011). All questions share a 5-point disagree/agree scale, which have been recoded so that right-wing/conservative attitudes indicate higher values (See Supplementary Material for full wording).

I measure *ideological distance* as the absolute value of the difference in attitude scores in the 5-point scale between the respondent's own position and the mean position of their out-party, which is itself calculated as the mean position of that party's voters. I then sum the ideological distance scores across all four questions. In operationalizing *party-issue constraint*, I first calculated the fraction of issue positions that are on either the left or right-wing side of the ideological divide. Scores below the overall mean (calculated across all respondents) are on the left and those above it, on the right. I then subtracted the fraction of right-wing positions from the fraction of left-wing positions (for PT voters), and vice-versa (for PSDB/PSL voters). For example, a value of 1 would mean that a PT voter held only left-wing views, whereas a value of −1 would indicate an ideologically left-wing respondent voted for the right-wing partisan option.^13^

*Social sorting* reflects how ‘sorted’ into their own partisan a camp a respondent is in terms of social demographics (sex, race, income, education, religion and region of residence). For each of these categories I calculate the proportion of respondents of the same demographic group as the respondent who voted for the respondent's party. As an example, 70% of Evangelicals in the BES 2018 sample voted for Jair Bolsonaro, meaning that an Evangelical Bolsonaro voter would receive a score of 0.7 on a ‘religious sorting’ variable. I repeat this procedure for the other six social demographic dimensions and calculate a mean social sorting score.

##### Control variables

I control for age, vote choice (‘PT Vote’ dummy), political interest (ranging from low to high self-declared interest in politics) and survey-year (with 2014 as baseline). I do not control for socio-demographic variables that are used in the calculation of social sorting. However, I run alternative models where these are included, e.g. Tables A3 and A16 in the Appendix.

### Results

**Figure 1.**
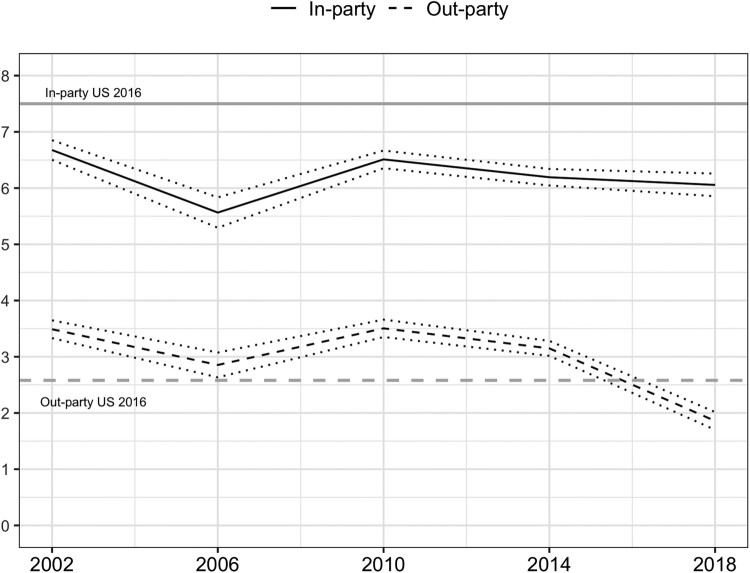
Affective ratings of in- and out-party in Brazil, 2002–2018. Notes: Affective ratings on 0 (dislike) to 10 (like) scale. Brazil data from BES 02-18, US data from CSES Module V. In- and out-party defined based on 2nd round presidential vote choice. Sample weights applied. Dotted lines represent 95% confidence intervals.

Though inconsistency in question-items restricts inferential analyses to 2014 and 2018, we can leverage past BES waves for some descriptive data. Figure 1 shows how in- and out-party ratings have changed since 2002 in Brazil. As a frame of reference, I include the same measures for the United States in 2016, a context recognized as being deeply polarized, using data from the Module 5 of the CSES. After remaining mostly constant between 2002 and 2014, out-party ratings plummeted in 2018 whilst in-party ratings saw no significant changes, serving as further circumstantial evidence that hating ‘the other’ is only weakly related to how much Brazilians like their own side. It is noteworthy that even this imperfect party-based measure still manages to capture the significant rise in out-group hostility at the 2018 elections, with Brazilians disliking their out-party significantly more than Americans did in 2016.

**Figure 2.**
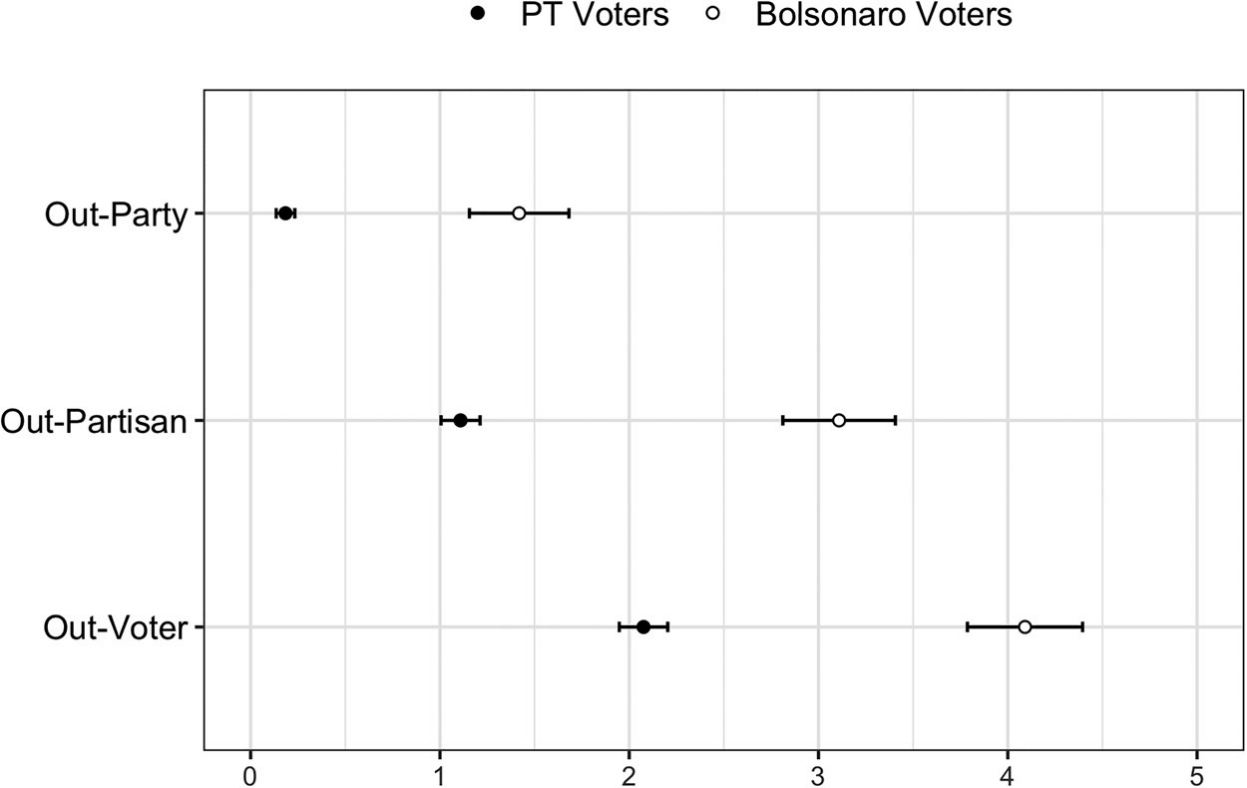
Online survey — affective rating of political out-groups. Notes: Affective ratings on 0 (dislike) to 10 (like) scale. Ratings of Jair Bolsonaro and his supporters were taken, respectively, as ‘Out-Party’ and ‘Out-Partisan’ measures for PT voters. Error bars indicate 95% confidence intervals.

Figure 2 displays the affective ratings assigned by survey respondents to their out-party (or out-candidate, for PT voters), out-partisans and out-voters. Note that for these results the affective scale is in its original form, with higher values indicating higher affect. In line with Druckman and Levendusky (2019) and Kingzette (2021), respondents clearly differentiated between voters, partisans and parties, with out-voters receiving higher affective scores. Additionally, PT voters consistently return lower affective evaluations of their political out-group, regardless of self-reported levels of political interest. Out-party dislike, the traditional measure of affective polarization in extant literature, is correlated at only 0.56 with out-partisan dislike, and even lower at 0.45 with out-voter dislike. In other words, inter-citizen hostility is only moderately captured by affective ratings of political elites.

I provide more descriptive findings related to key variables in the Appendix. More importantly, 53% of Bolsonaro voters agreed or heavily agreed that they would describe themselves as ‘anti-PT’, and around 19% described themselves as *bolsonaristas*. For PT voters, 88% described themselves as ‘anti-Bolsonaro’, but only 8% as *petistas*. In sum, negative identities were much more prevalent than positive ones, especially amongst PT voters. These findings remained the same if using the entire PID and NID scales (i.e. summing the individual items and taking the overall mean for each scale), and across levels of self-reported political interest.

#### Model results

**Figure 3.**
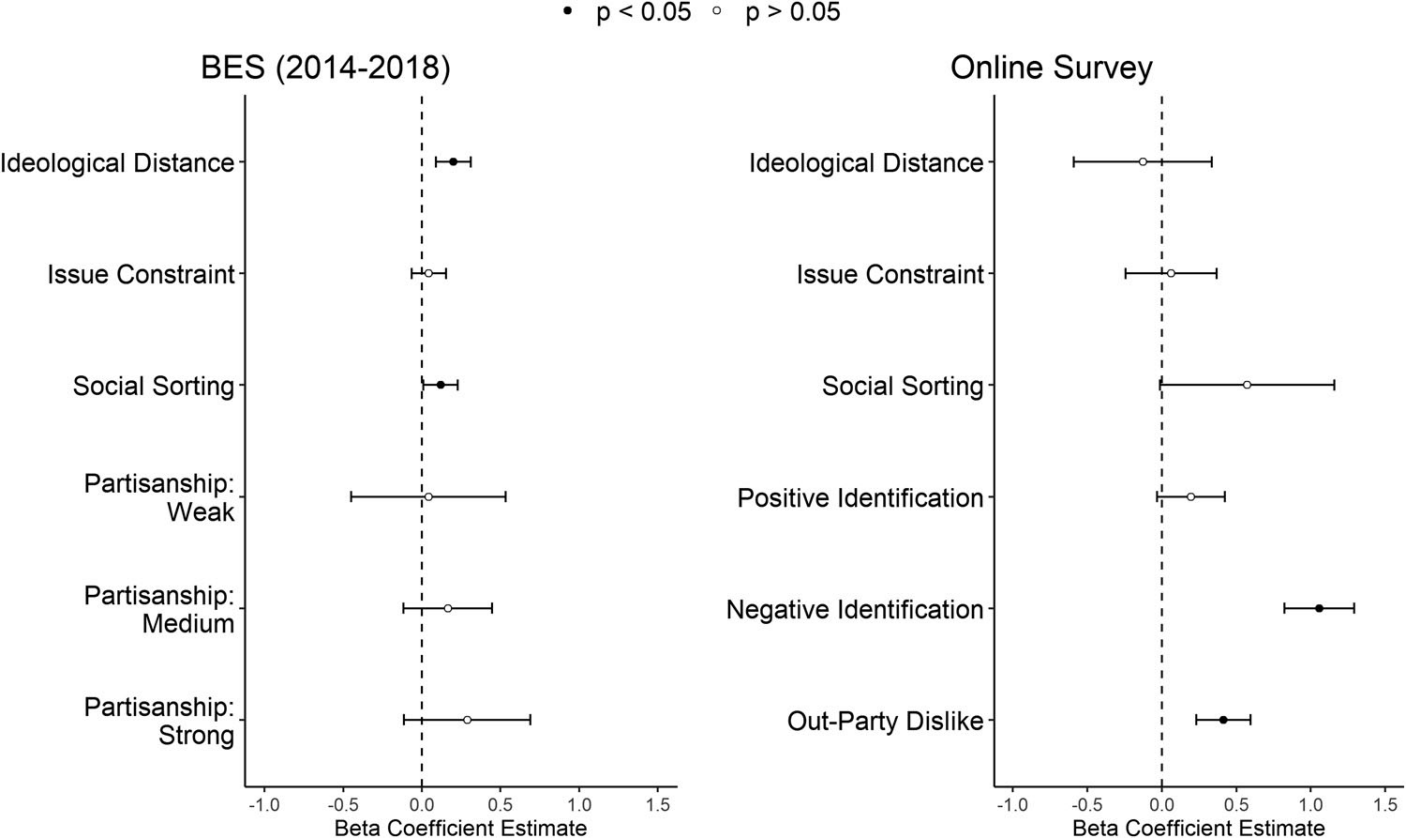
OLS model results. Notes: BES: *N* = 2870. DV: Out-Party Dislike. Online Survey *N* = 865. DV: Out-Voter Dislike. 95% confidence intervals calculated from robust standard errors. Sample weights applied. Control variables not shown. See Appendix for full regression tables.

Figure 3 displays coefficient plots of OLS models for the BES and online survey data. Control variables are not displayed (see A2 and A17 in Appendix for full regression table). For the BES model the dependent variable is the reverse coded affective rating attributed to the respondent's out-party on a 0–10 scale (i.e. party they did not vote for in the second round of the presidential election), whilst in the online survey model I use affective ratings of out-voters. All numerical independent variables (i.e. ideological distance, issue constraint, and social sorting) are standardized into z-scores, such that their coefficients should be interpreted as the predicted effect of an increase of one standard deviation on the 0–10 affective scale. Sample weights provided by the BES dataset were applied, while weights based on the BES population data were used for the survey model. Error bars indicate a 95% confidence interval calculated from robust standard errors.

Hypothesis 1a posited that those ideologically distant from ‘the other side’ should display higher levels of affective polarization. In the BES model this association is statistically significant (β=0.20, *p* = 0.000), but in the opposite direction and without reaching statistical significance in the survey model (β=−0.12, *p* = 0.36). Whilst these results are inconclusive, even in the BES model the effect of ideological distance is substantively small. Varying ideological distance across its interquartile range (i.e. 25th to 75th percentile) whilst holding every other variable at their mean only increases the predicted value of out-party dislike by 0.3 points in the 11-point affective scale, or 0.10 standard deviations. Further, the effects of issue constraint (Hypothesis 1b) in both models is minimal and statistically insignificant. Put together, these findings highlight the weak explanatory power of ideology-based mechanisms in explaining affective polarization in Brazil. To the extent that ideology does drive out-group hostility, this seems to happen not via respondents' alignment with their own ‘side’, but rather through being distant from ‘the other’. Indeed, this is line with the general weakness of in-party attachments and the weak association between ideology and party support in Brazil.

Hypothesis 2a predicted that a strong sense of identification with one's in-party should predict higher levels of out-group hostility. Initial results from both models do not find evidence for this hypothesis, which was tested via a partisanship strength variable (BES) and the Partisan Identity Scale (Online Survey). As previously mentioned, however, the full PID scale contains two items with relatively poor factor loadings in their theorized dimension, which also load somewhat strongly in the NID dimension. This may obfuscate the independent effects of positive identification. To remedy this issue I ran an alternative model (Table A17 in Appendix) using only the fourth items from the NID and PID scales (i.e. ‘I would describe myself as (anti)…’). These items had strong factor loadings in their respective dimensions, have directly comparable wording, and are only weakly correlated (Pearson's *r* = 0.15). In this model, positive identification returns a statistically significant association with out-voter dislike (β=0.24, *p* = 0.000). In line with Hypothesis 2a, this alternative model suggest that positive political identities do play a role in fostering affective polarization in Brazil.

As per Hypothesis 2b, socially sorted individuals should display higher levels of affective polarization due to overlapping social identities. This is indeed the case in the BES model (β=0.119, *p* = 0.03), though in the survey model the coefficient is substantively large but not statistically significant (β=0.59, *p* = 0.09). This estimate, however, is rather uncertain as evidenced by its wide confidence interval. A possible explanation for this lies in the weighting procedure applied to the survey model, with weights being calculated by the same demographic variables used to calculate a final measure of social sorting. Results from the BES model provide a more robust test of this particular variable, and the significant effect of social sorting is in line with the increased demographic polarization found by Layton et al. (2021).^14^

The key independent variable in the main survey model, negative political identity, returns a strong and statistically significant association with out-voter dislike. A one standard deviation increase in negative identification increases out-voter dislike by 0.86 on the 11-point dislike scale (*p* = 0.000). Across its interquartile range, negative identification increases out-voter dislike by 1.29 points, almost equal to a full standard deviation on the outcome variable (SD=1.32) It is noteworthy that such strong and robust effect is found even with the inclusion of out-party dislike in the model, which itself returns a significant and substantive result (β=0.57, *p* = 0.000). A valid counter-argument here is that this result is tautological, as the negative identity scale directly mentions the group being evaluated as the dependent variable. However, in a model foreshadowed above where only the single ‘I would describe myself as anti [out-party]’ item is used, this strong effect remains. This item makes no mention of out-voters and directly taps into a sense of identification, arguably providing a more robust test of Hypothesis 3. This result suggests that negatively identifying with the out-party/leader increases out-voter dislike even when accounting for an instrumental dislike of one's out-party/leader. This also further confirms the differentiation between negative evaluations and negative identities: people do not ‘negatively identify’ with everything they dislike (Bankert 2021), and there is an independent and sizeable effect of having such negative identities.

Hypothesis 3 was specifically about whether the effect of negative identities on affective polarization is stronger than that of mere positive identification. While this is obviously the case in the main survey model with the ‘noisy’ PID scale, I further analyse the model that uses only the single-item PID and NID measures. In this model both variables were statistically and substantively significant in predicting affective polarization. Negative identification still returned a stronger effect (β=0.78, *p* = 0.000) than positive identification (β=0.27, *p* = 0.01), which was confirmed by a robust *F*-test for the difference in coefficients between these two variables. The results confirm that negative identification has a statistically significant stronger effect size on out-voter dislike, in line with Hypothesis 3 (*F* = 8.73, p=0.003).

#### The explanatory power of negative identities

A central argument in this paper is that ‘traditional’ explanations for affective polarization are unlikely to properly account for the phenomenon in Brazil. While these mechanisms could work at the individual-level, I expected them to hold little explanatory power. Formally, this could be analysed by gauging the variance of the dependent variable that is explained by the model predictors. In the table below I compare the adjusted-$R^{2}$ (which accounts for variance-inflation as a result of new variables being added across different models) of the BES model and different iterations of the Online Survey model.

The full BES model, which tests hypotheses related to ideology and (positive) social identities, returns an adjusted-$R^{2}$ of only 0.08, meaning that only 8% of the variation in affective polarization can be explained by the predictors (accounting for the number of predictors inserted into the model). Put simply, the BES does very poorly in accounting for affective polarization in Brazil.

**Table 3.** Explained variance of survey OLS models.

	BES Full model	Survey base model	Base model + PID	Base model + NID	Base model + PID + NID	Survey full model
Adj-$R^{2}$	0.08	0.13	0.21	0.40	0.40	0.42

The *survey base model* ‘mirrors’ the BES model in containing ideological distance, issue constraint and social sorting, variables that were operationalized in the same way across these models. The 0.13 adjusted-$R^{2}$ indicates that, even without partisanship, this iteration outperforms the BES model. This difference may lie on different operationalisations of the dependent variable (out-party for BES, out-voter for Online Survey) or sample bias, and thus this model primarily serves as the baseline for other iterations. Adding the PID measure to the baseline model increases the adjusted-$R^{2}$ to 0.21. More importantly, however, is that adding negative identification to the base model (i.e. without PID) results in a much higher adjusted-$R^{2}$ of 0.40. Once PID is included in this model, there is no change in the adjusted-$R^{2}$. Finally, the full model adds the *out-party dislike* variable, which served as a hard constraint on negative identification, resulting in the final adjusted-$R^{2}$ of 0.42.

In sum, negative identification has by far the strongest explanatory value in predicting affective polarization in Brazil. Given the weak explanatory power of other independent variables, these results strongly suggest that negative identities are, indeed, a much better explanation for affective polarization in Brazil than those following from mainstream hypotheses.

## Conclusion

What explains affective polarization in Brazil? The findings presented here suggest that explanations widely applied to the contexts of the United States and Western Europe cannot satisfactorily account for why Brazilians of different side of politics feel hostile towards each other. Ideological distance, alignment between ideology and party of choice, and social divisions are only inconsistently associated with affective polarization, providing very little explanatory power. Similarly, Brazilians do not seem to like or identify with their own ‘side’ to any significant degree. The evidence presented here tentatively points towards a still overlooked and underdeveloped concept in political science: negative political identities. A sense of negative identification with one's out-party or leader is significantly and substantively correlated with high levels of dislike towards out-voters. Whilst affective polarization is often framed as a battle between ‘us’ versus ‘them’, Brazilians seem to dislike ‘them’ without having or particularly liking any clearly defined ‘us’.

The effects of these negative identities are not restricted to a mere dislike of the out-group. Past research has shown that negative identities can hamper cross-party cooperation (Bankert 2021) and satisfaction with democracy when the opposition is in power (Spoon and Kanthak 2019), spelling a worrying future for the quality and stability of Brazilian democracy. On the other hand, positive outcomes might emerge as corollary of this increase and strengthening of negative political identities. Disliking the other side can act as an important motivation for political participation (Caruana, McGregor, and Stephenson 2015). The mass protests of recent years indicate politics is increasingly salient to Brazilians, who had previously shown low and decreasing levels of engagement with political activities (Borba and Ribeiro 2018). In addition, out-party cues can be just as strong as in-party cues in influencing individuals' policy views (Samuels and Zucco 2018), such that negative identities can mirror partisan attachments (Levendusky 2010) in inducing greater constraint in the ideologically inconsistent Brazilian electorate.

The theoretical and empirical findings of this paper go well beyond the particular case of Brazil. In an era of weakening partisan ties (Dalton and Wattenberg 2002), negative partisanship is an increasingly relevant factor determining political behaviour in contexts such as the US (Abramowitz and Webster 2016), Canada (Caruana, McGregor, and Stephenson 2015), and across Anglo (Medeiros and Noël 2014) and European (Mayer 2017) democracies. This literature, however, still suffers from the lack of an integrated theoretical framework and robust conceptualization. The theoretical discussions and empirical measures of negative identities proposed in this paper can thus be of guidance for future research. Much like its positive counterpart, negative partisanship can also be conceptualized and measured as a type of social identity, exerting strong independent effects on political attitudes. Future studies could explore the observable implications of this conceptualization, namely information-seeking behaviour, motivated reasoning, and stability of identification. Further, the case of Brazil suggests that factors such as weak party system institutionalization, non-programmatic and dichotomized competition, and low levels of political trust may foster the formation of negative identities. Future studies should thus provide a systematic and empirical examination of these potential explanations, as well as explore broader consequences of political identities in a comparative framework.

There are, however, important limitations that warrant caution in understanding the determinants of affective polarization in Brazil. The measurement approach taken to assess the effects of ideology-based explanations infers both distance and alignment from aggregate measures, and does not directly expose individuals to different views held by their fellow citizens. Further studies could achieve this through an experimental design. Similarly, measures of social sorting captures objective social demographics rather than an individual's subjective sense of identification with his or her social category (see Mason and Wronski 2018), which future research should also address. Thirdly, though several measures were taken to account for the less-than-ideal survey sample, we cannot fully discard that findings might be driven by the skewness of the sample towards individuals of higher socio-economic status. Though robust to several different specifications and a hard set of controls, the effects of negative identities on out-group hostility can be better assessed by, for instance, priming subjects' need for distinctiveness through an experiment, with a view to both confirming the mechanism and causally testing its effects. Lastly, future research can also study the effects of the media environment on affective polarization, an angle not explored here.

In spite of these shortcomings, this paper meaningfully inserts the case of Brazil in the broader literature on affective polarization, highlighting the need to study the concept in political contexts that go beyond those that tend to attract the bulk of scholarly interest. Traditional explanations might tell us little about the roots of affective polarization beyond the United States and Western Europe. From Brazil to Poland, from Kenya to India, political divides are becoming increasingly hostile (Carothers and O'Donohue 2019). It is therefore imperative that political scientists continue developing a comparative approach in understanding what turns politics into a nasty game between enemies.

## Supplementary Material

Supplemental Material

## Notes

1 This differs from initial definitions that conceptualized affective polarization as the tendency of partisans to see co-partisans positively and out-partisans negatively (Iyengar, Sood, and Lelkes 2012), since this imbues the notion of out-group hostility with in-group bias. Including in-party bias in the definition of affective polarization would obfuscate the prevalence of out-group hostility in contexts, such as Brazil, of low partisan attachments.

2 To be clear, the point is not that those mechanisms are wrong at the individual-level, but rather that they would explain very little of aggregate-level affective polarization in Brazil due to low scores on the independent variables.

3 Scholars such as Lee et al. (2022) and Rosema and Mayer (2020), for instance, argue that negative partisanship is restricted to negative evaluations rather than a acting as a social identity akin to positive partisanship.

4 Though see Medeiros and Noël (2014), Abramowitz and Webster (2018), and Anderson, McGregor, and Stephenson (2021).

5 Note, however, that a positive or negative identity around the PT is conceptually different from evaluations of the PT's main historical leader, former president Lula da Silva. Feelings towards Lula are closer to retrospective evaluations based on economic performance rather than a sense of identification (Samuels and Zucco 2014b).

6 Though see Almeida Resende and Chagas-Bastos (2021)'s argument that antipetismo derives from diverging opinions on the military dictatorship.

7 Please see Supplementary Material for full wording. The procedure followed the guidelines of the Amsterdam Institute for Social Science Research (AISSR).

8 Funds were provided by the Dutch Research Council (016.Veni.195.159).

9 A potential issue with this approach is that the BES samples only city-dwelling Brazilians, which compromise around 85% of the population (IBGE 2016). As a robustness check, I run the main model using weights based on the 2010 Brazilian Census (Table A13). Results are not substantially different.

10 In practice, a weight of 3 means the respondent would count 3 times as much as their original value. Alternative upper and lower bounds did not produce substantively different results (see Table A14).

11 See Table A4 for first-round only results.

12 See Table A15 for a model with out-partisan dislike as dependent variable.

13 This strategy is inspired by Mason (2018a).

14 However, I find no evidence that Evangelical individuals are more affectively polarized. See Table A6.
