# Supplementary figures and images for: ‘Them’ without ‘us’: negative identities and affective polarization in Brazil
Source: Political Res Exch. 2022 Sep 5;4(1):2117635. doi: 10.1080/2474736X.2022.2117635 (PMC9484554; doi:10.1080/2474736X.2022.2117635)

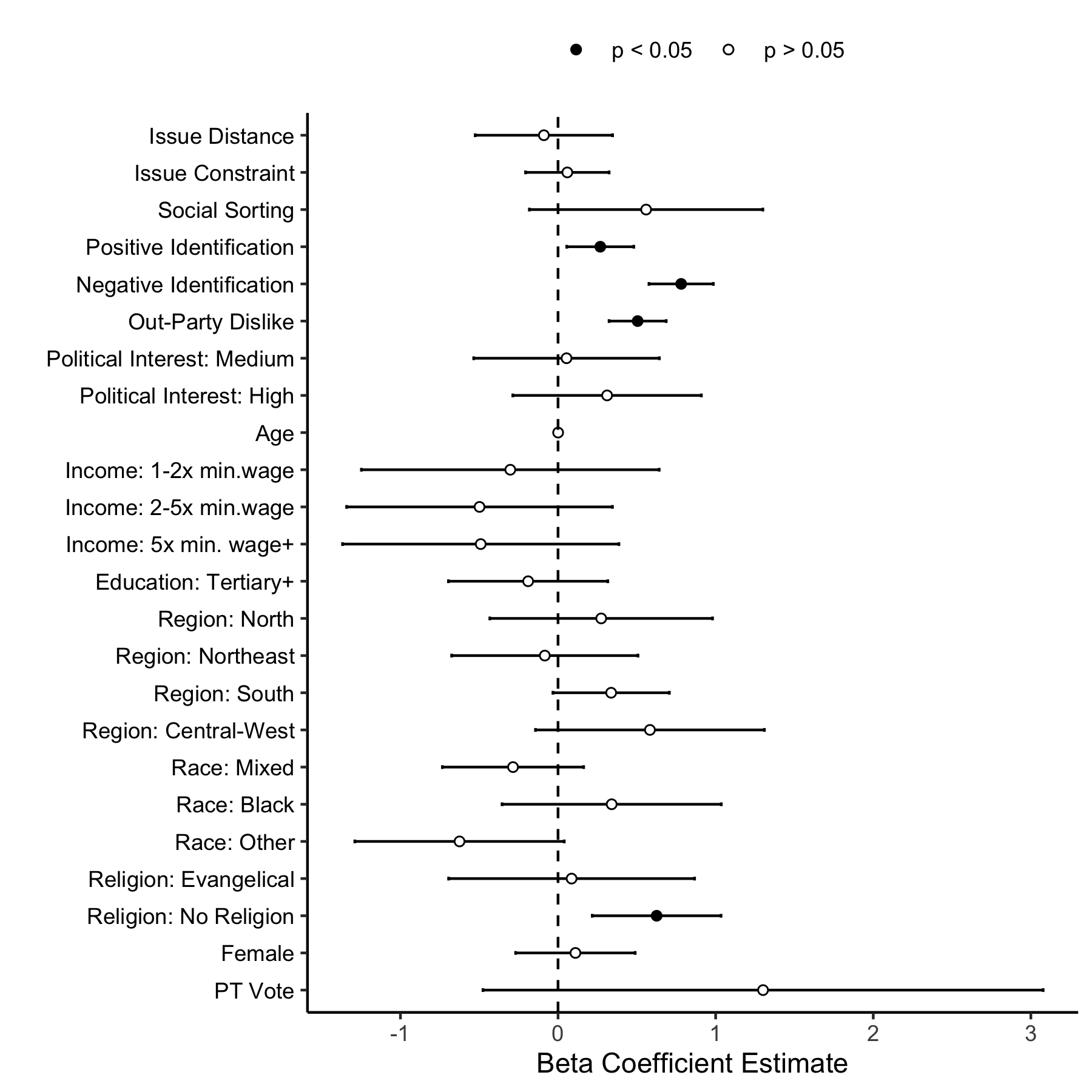

Supplement: Supplemental Material [file PRXX_A_2117635_SM2768.zip › mainsurveymodel.png]

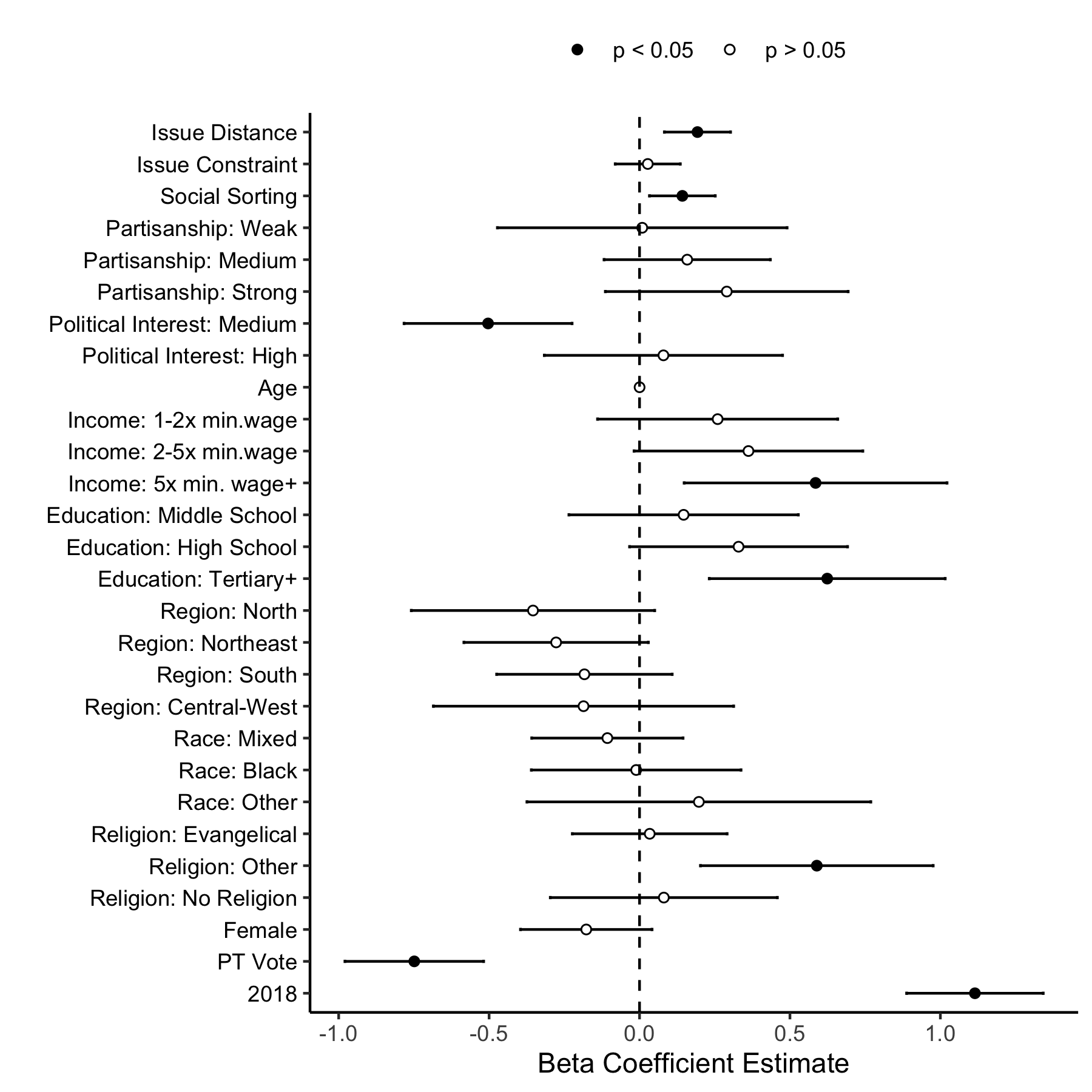

Supplement: Supplemental Material [file PRXX_A_2117635_SM2768.zip › besmodel.png]

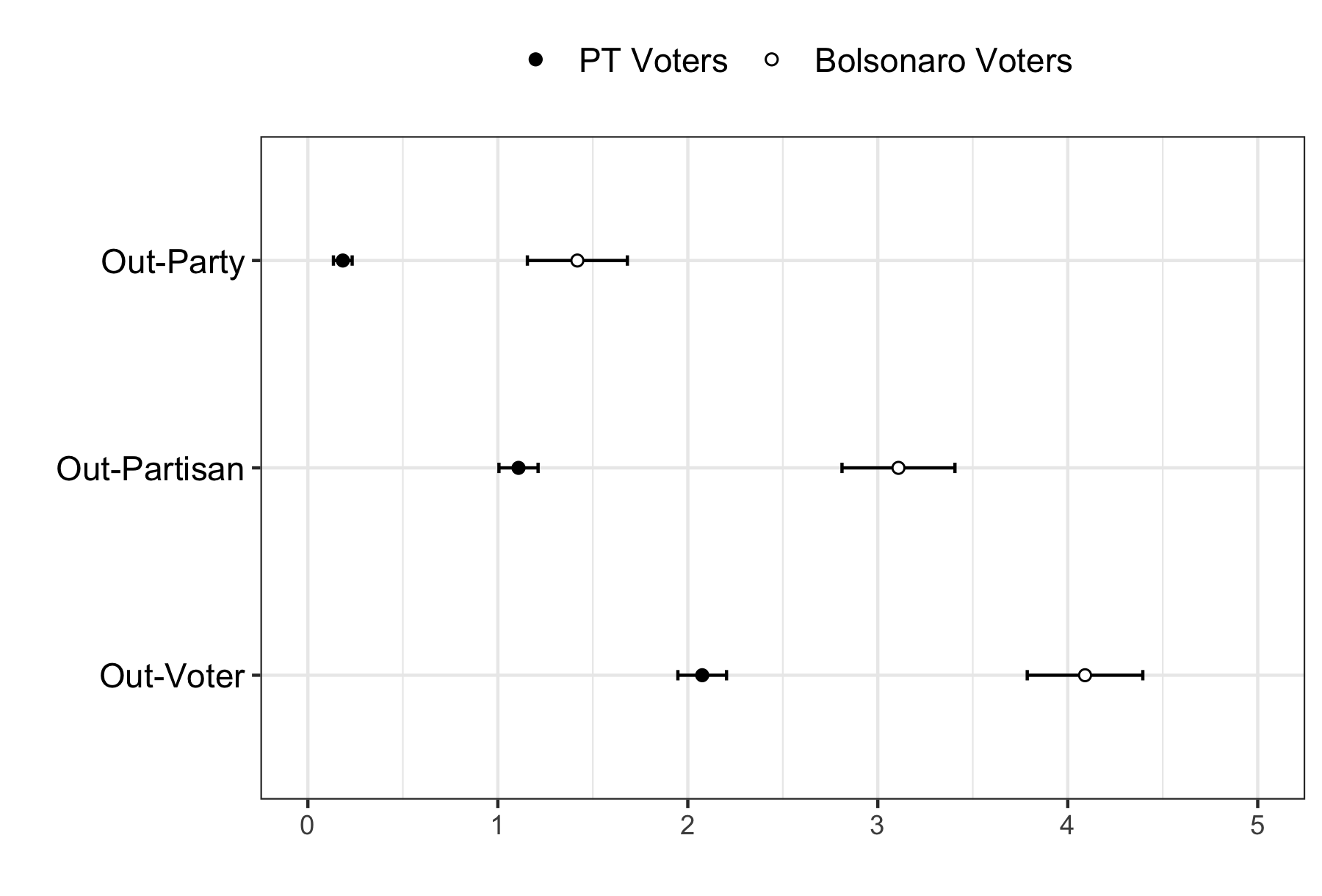

Supplement: Supplemental Material [file PRXX_A_2117635_SM2768.zip › outcome.png]

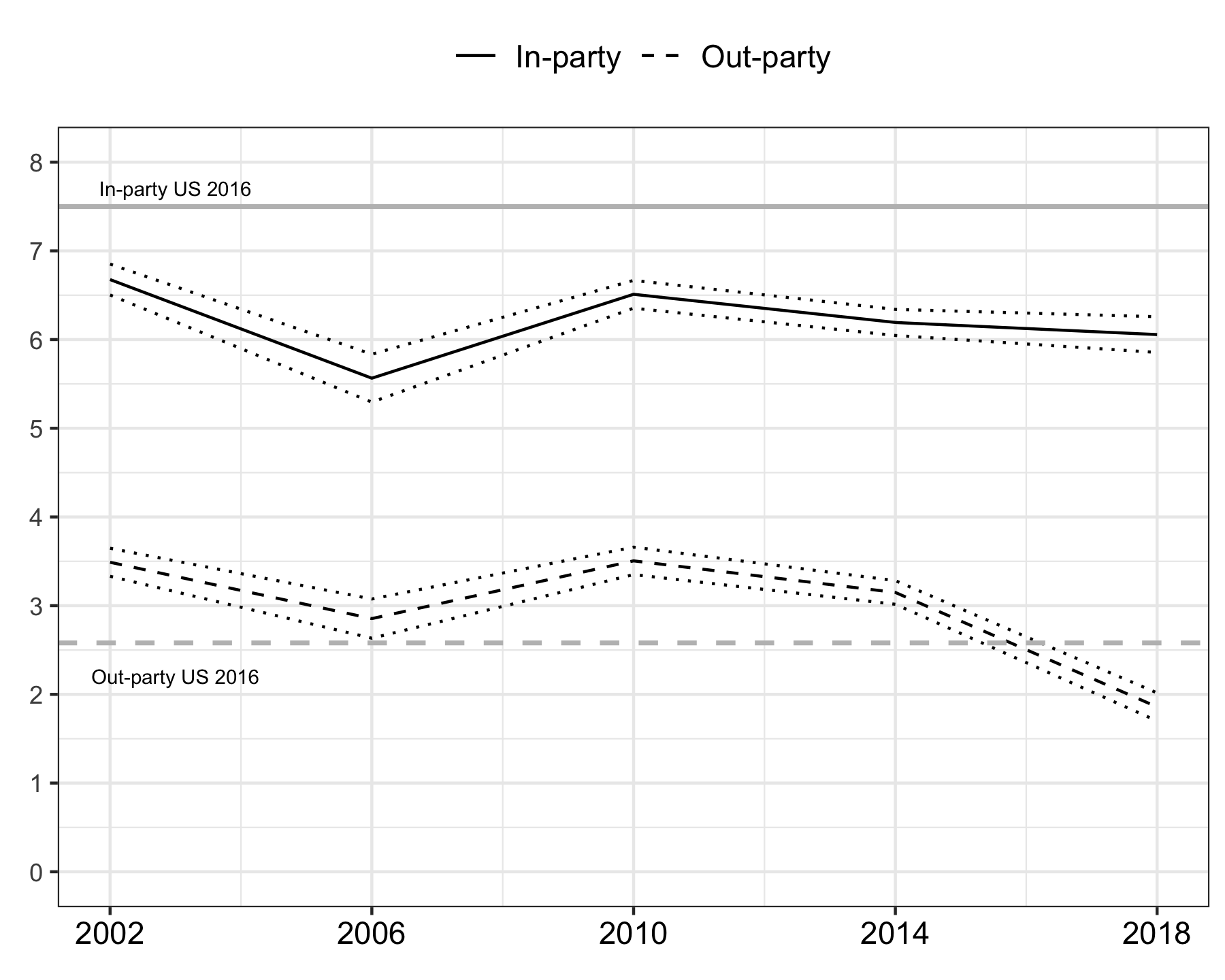

Supplement: Supplemental Material [file PRXX_A_2117635_SM2768.zip › affpol.png]

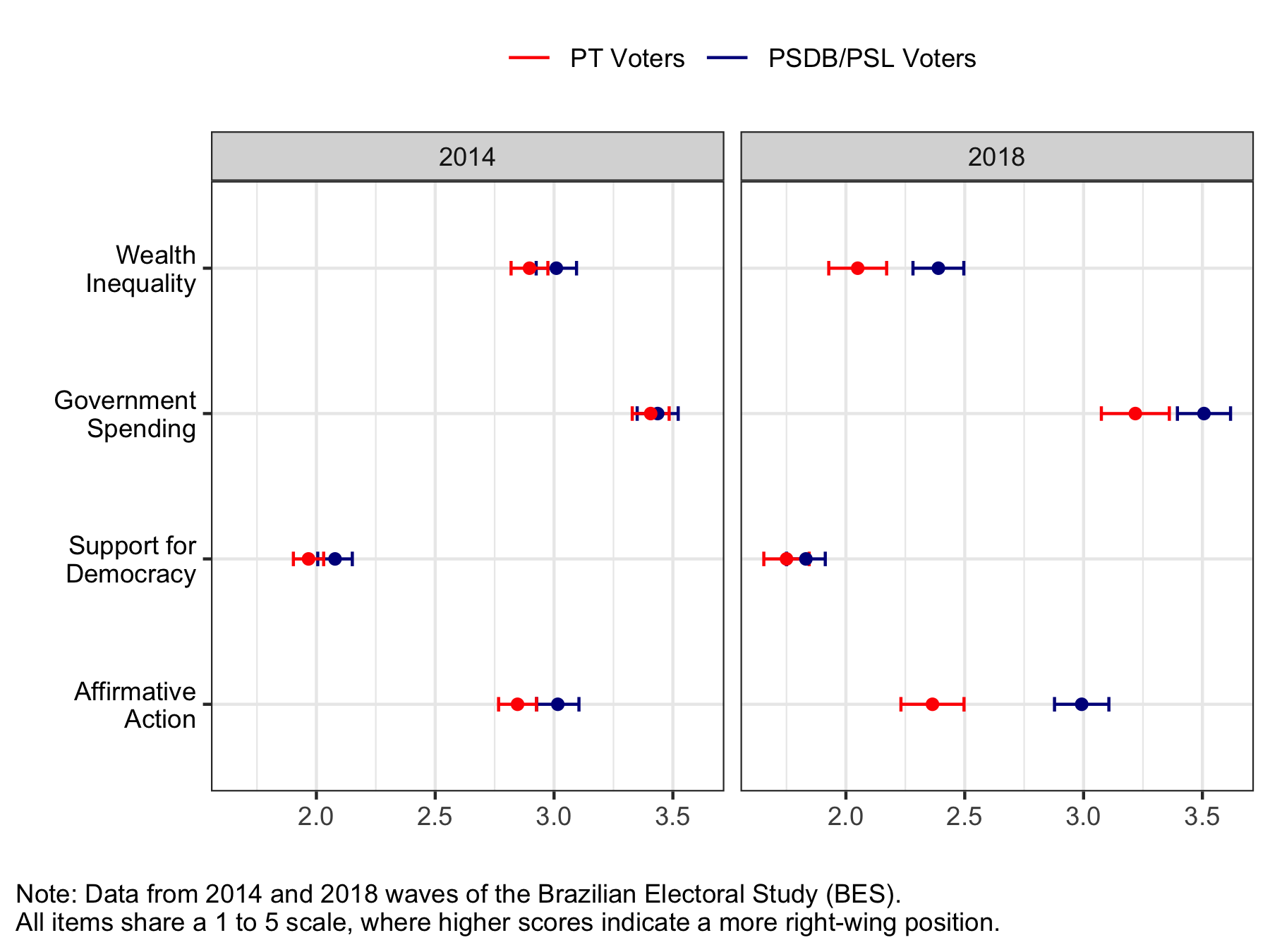

Supplement: Supplemental Material [file PRXX_A_2117635_SM2768.zip › issuepolarisation.png]

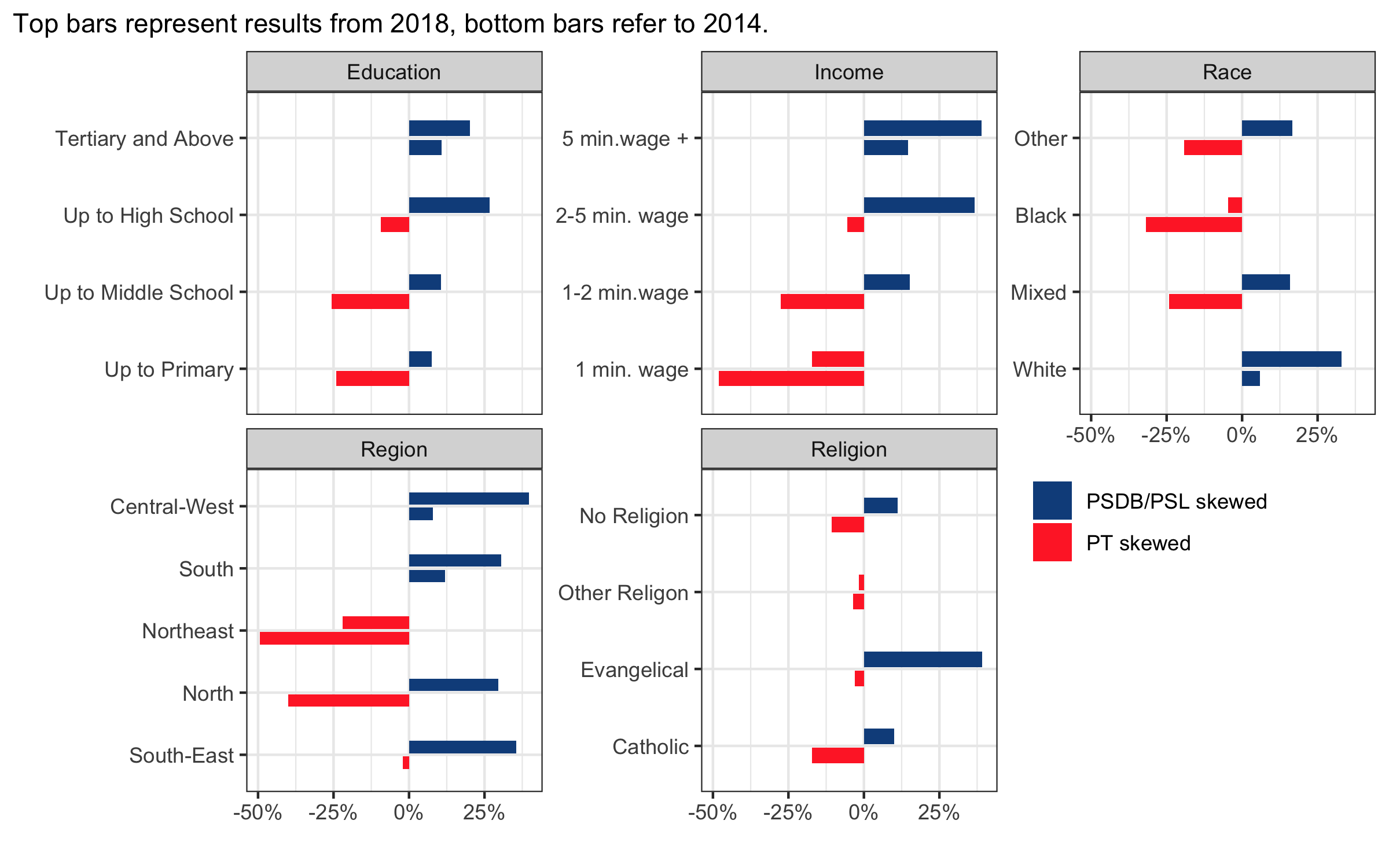

Supplement: Supplemental Material [file PRXX_A_2117635_SM2768.zip › socialsorting.png]

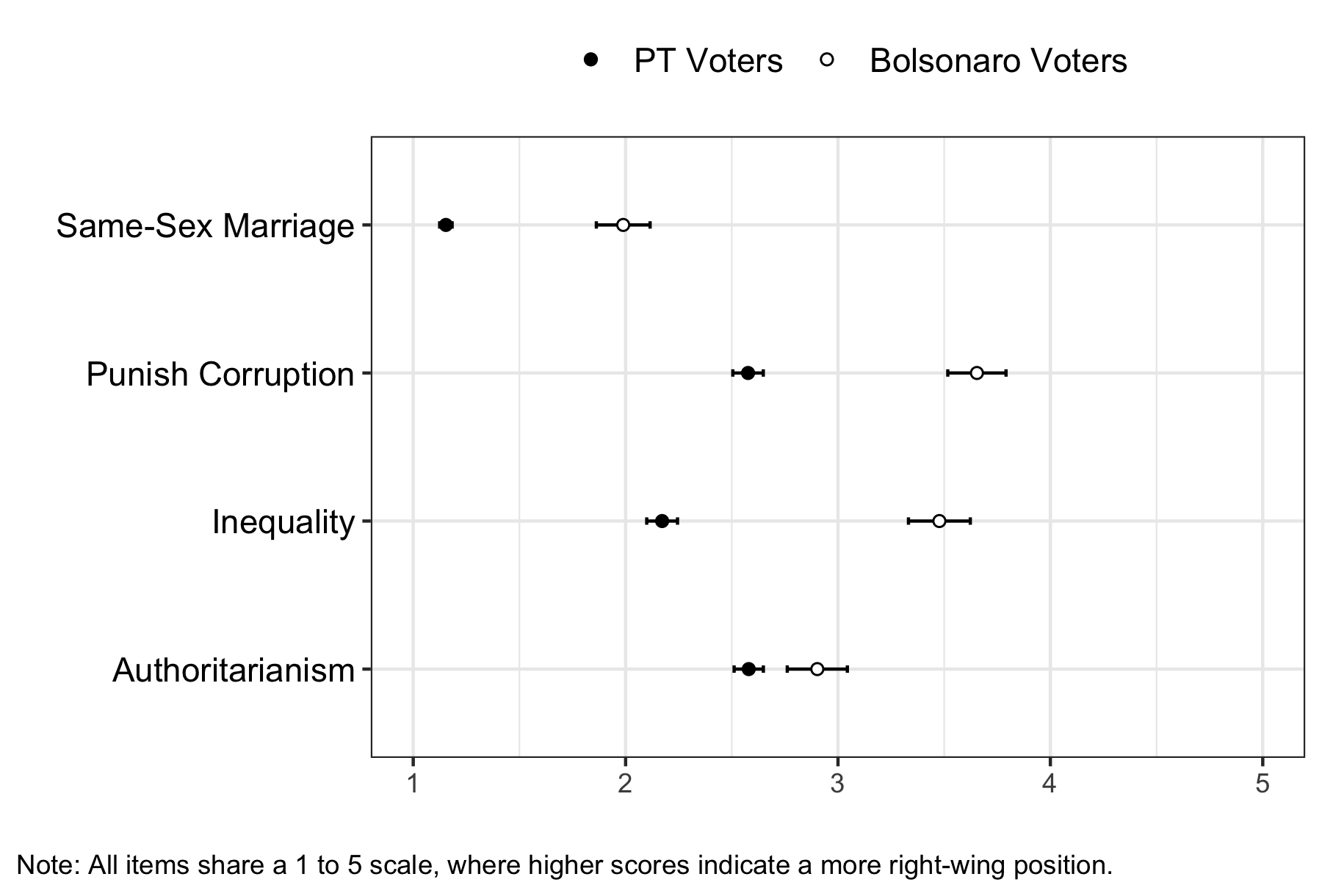

Supplement: Supplemental Material [file PRXX_A_2117635_SM2768.zip › svyissuepol.png]

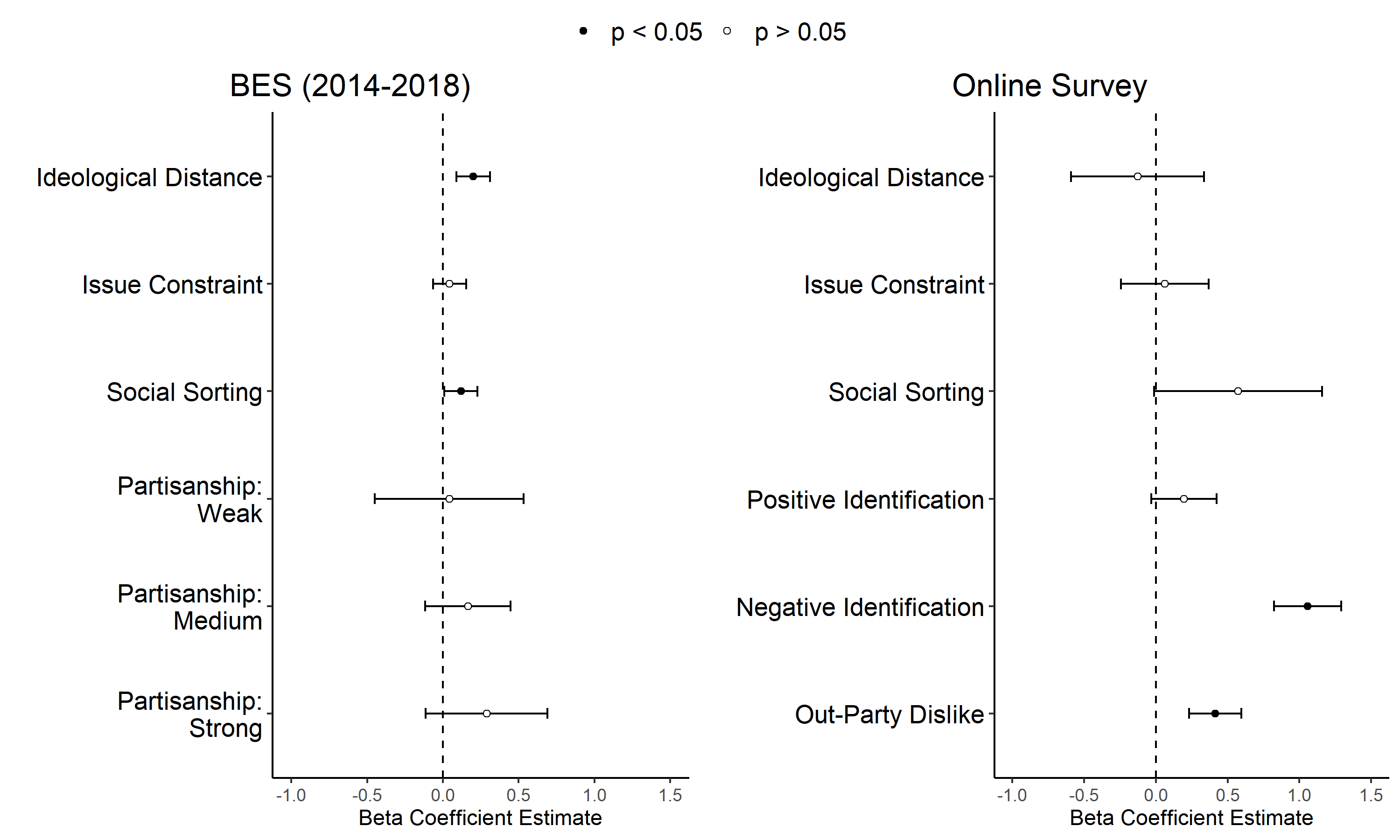

Supplement: Supplemental Material [file PRXX_A_2117635_SM2768.zip › models_rewrite.png]

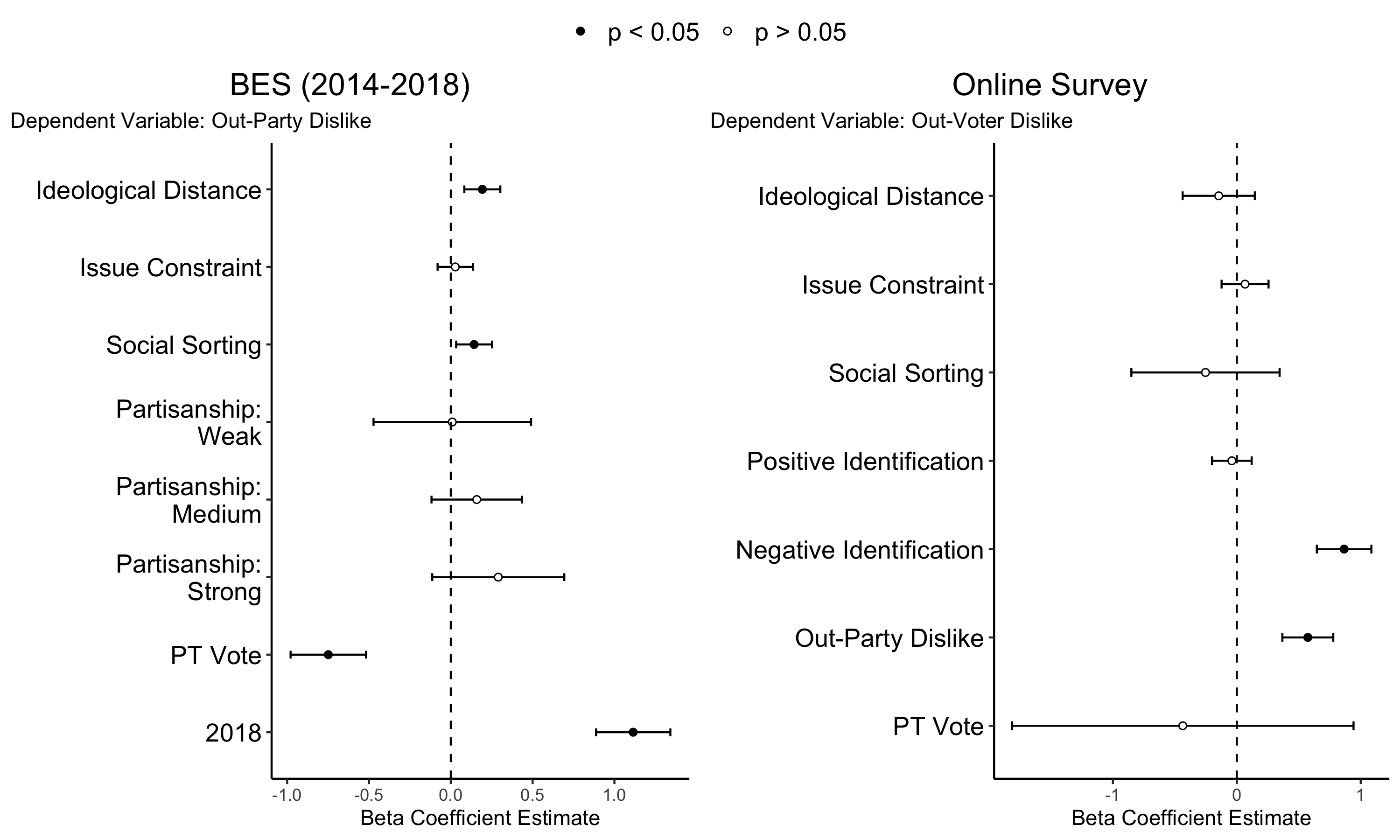

Supplement: Supplemental Material [file PRXX_A_2117635_SM2768.zip › models_ggsave.png]

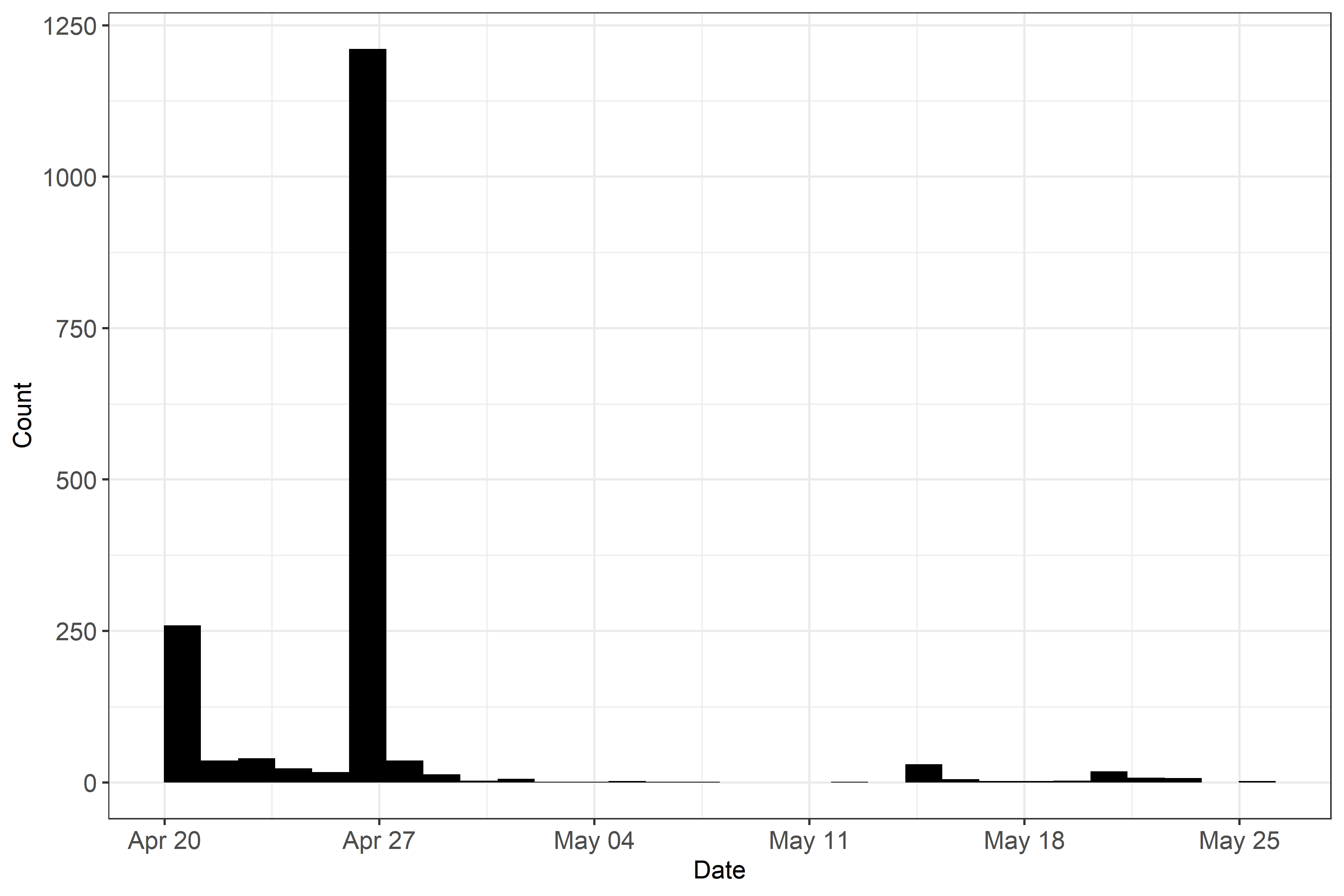

Supplement: Supplemental Material [file PRXX_A_2117635_SM2768.zip › dist_survey.png]
